# Cerebrospinal fluid‐specific oligoclonal bands in dogs with idiopathic epilepsy

**DOI:** 10.1111/jvim.17265

**Published:** 2024-12-23

**Authors:** Junwei Föhr, Julia K. Prümmer, Arianna Maiolini, Eliane Marti, Ilijas Jelcic, Beatriz Vidondo, Mario Ziegler, Andrea Bathen‐Nöthen, Andrea Tipold, Holger A. Volk, Veronika M. Stein

**Affiliations:** ^1^ Division of Clinical Neurology, Vetsuisse Faculty University of Bern Bern Switzerland; ^2^ Division of Neurological Sciences, Vetsuisse Faculty University of Bern Bern Switzerland; ^3^ Department of Neurology University of Zurich Zurich Switzerland; ^4^ Department of Clinical Research and Public Health, Vetsuisse Faculty University of Bern Bern Switzerland; ^5^ Veterinary Practice Dr. A. Bathen‐Nöthen Cologne Germany; ^6^ Department of Small Animal Medicine and Surgery University of Veterinary Medicine Hannover Hannover Germany

**Keywords:** ASM resistance, autoimmune encephalitis, canine idiopathic epilepsy, neuroinflammation, oligoclonal bands

## Abstract

**Background:**

In dogs with idiopathic epilepsy (IE), 33% develop resistance to conventional anti‐seizure medication (ASM) despite adequate treatment. In human medicine, an immune‐mediated etiology is suspected in a subset of ASM‐resistant patients with epilepsy and cerebrospinal fluid (CSF)‐specific immunoglobulin G (IgG)‐type oligoclonal bands (OCBs) have been detected. In dogs, cases of autoimmune encephalitis recently were reported. Neuroinflammation may provide an additional explanation for the lack of response of certain dogs with IE to ASM.

**Hypothesis:**

Cerebrospinal fluid‐specific OCBs are found in a subgroup of dogs with ASM‐resistant IE.

**Animals:**

Eighty‐four dogs with IE were recruited from 3 referral centers and classified based on their response to ASM treatment (responsive, n = 56; resistant, n = 28).

**Methods:**

Detection of OCBs was performed using isoelectric focusing (IEF) followed by immunoblotting. Associations of CSF‐specific OCBs with seizure type, severity, and response to ASM were calculated using logistic regression models.

**Results:**

The overall frequency of CSF‐specific OCBs in dogs with IE was 15.5% (95% confidence interval [CI], 8.5%‐25%). In dogs with ASM‐resistant IE, 21.4% (6/28) had CSF‐specific OCBs compared with only 12.5% (7/56) in those responsive to ASM, but no evidence of an association was detected (odds ratio, 1.9; 95% CI, 0.57‐6.35; *P* = .29).

**Conclusions and Clinical Importance:**

Cerebrospinal fluid‐specific OCBs were detected in a subgroup of dogs with IE. This finding could indicate that intrathecal IgG synthesis as a sign of neuroinflammation may play a role in disease pathogenesis.

**Abbreviations**
anti‐LGI1: anti‐leucine‐rich glioma inactivated 1
anti‐NMDAR: anti‐N‐methyl‐D‐aspartate receptor
anti‐GABA_A_R: anti‐r‐aminobutyric acid type A receptor
anti‐GABA_B_R: anti‐r‐aminobutyric acid type B receptor
APE: antibody prevalence in epilepsy
ASM: anti‐seizure medication
CASPR2: contactin‐associated protein‐like receptor 2
CI: confidence interval
CL: confidence level
CNS: central nervous system
CSF: cerebrospinal fluid
ECVN: European College of Veterinary Neurology
ESVN: European Society of Veterinary Neurology
GAD: glutamic acid decarboxylase
IE: idiopathic epilepsy
IEF: isoelectric focusing
IgG: immunoglobulin G
IVETF: International Veterinary Epilepsy Task Force
n.k: not known
OCBs: oligoclonal bands
TNCC: total nucleated cell counts
TP: total protein

## 1 INTRODUCTION

Idiopathic epilepsy (IE) is the most common type of epilepsy in dogs.^1^ It is defined as “*2 or more unprovoked seizures at least 24 hours apart with no identifiable underlying etiology other than a suspected genetic origin*.”^2^ Idiopathic epilepsy is treated symptomatically with anti‐seizure medication (ASM), such as phenobarbital, imepitoin, potassium bromide or levetiracetam, as monotherapy or in combination. Although most dogs with IE show a decrease in seizure frequency, intensity or both, approximately 25%‐30% of dogs are resistant to conventional ASM.^2^, ^3^ In comparison, approximately 20%‐40% of human patients with epilepsy are estimated to be resistant to ASM.^4^ An autoimmune etiology has been recognized in some human patients with epilepsy^5^, ^6^, ^7^ and, in particular, in a subgroup of patients with ASM resistance.^8^ Affected patients may manifest seizures and other concomitant signs of autoimmune encephalitis, such as neuropsychiatric symptoms, short‐term memory loss, altered level of consciousness, lethargy, or personality change, suggesting involvement of the limbic system.^9^ The International League Against Epilepsy revised the classification and terminology in 2017 with the proposal for the new classification of autoimmune epilepsy as “*epilepsy with evidence of autoimmune mediated central nervous system (CNS) inflammation*.”^10^ Several different antibodies have been described to be involved in autoimmune encephalitis in human patients (eg, anti‐N‐methyl‐D‐aspartate receptor [anti‐NMDAR] antibodies,^11^ anti‐γ‐aminobutyric acid type B^12^ and A^13^ receptor [anti‐GABA_B_R and GABA_A_R] antibodies, anti‐leucine‐rich glioma inactivated 1 [anti‐LGI1] antibodies).^14^

In veterinary medicine, a possible Th17 immune‐mediated etiology has been identified in single cases of dogs diagnosed with IE, which was associated with a higher risk for cluster seizures and status epilepticus in affected dogs.^15^ Furthermore, the first case of anti‐GABA_A_R encephalitis recently has been described in a Cavalier King Charles Spaniel with continuous seizures and behavioral abnormalities despite adequate seizure management, which responded well to immunotherapy with dexamethasone.^16^

Immunoglobulin G (IgG) oligoclonal bands (OCBs) are found in the cerebrospinal fluid (CSF) of a variety of different neurological and non‐neurological diseases, but are best known in multiple sclerosis, a chronic inflammatory disease affecting the CNS of humans.^17^, ^18^ Two or more OCBs found in CSF, but not in serum of the respective patient (ie, CSF‐specific OCBs) indicate intrathecal synthesis of IgG, reflecting a local B‐cell response.^19^ In a recent study investigating OCBs in dogs, 1 patient in the IE group was found to have CSF‐specific OCBs. Interestingly, this patient was resistant to ASM.^20^ Our aim was to (a) detect the presence of CSF‐specific OCBs in a larger population of dogs diagnosed with IE and (b) investigate a possible association between the presence of CSF‐specific OCBs and response to ASM. We hypothesized that CSF‐specific OCBs would be found in a subgroup of dogs with ASM‐resistant IE.

## 2 MATERIALS AND METHODS

### 2.1 Dogs

For the retrospective multicenter study, dogs presented from 2016 to 2022 for diagnostic evaluation of epileptic seizures were recruited from 3 referral centers: Division of Clinical Neurology, Small Animal Clinic, Vetsuisse Faculty, University of Bern, Switzerland, the Veterinary Practice Dr. A. Bathen‐Nöthen, Cologne, Germany, and the Department of Small Animal Medicine and Surgery, University of Veterinary Medicine Hannover, Germany. All dogs were examined by a board‐certified neurologist and diagnosed with IE according to the current guidelines of the International Veterinary Epilepsy Task Force (IVETF), tier II confidence level. All of the included dogs had a minimum follow‐up period of 4 months. Dogs were assigned to the following 2 groups: ASM‐resistant IE and ASM‐responsive IE. Resistance to ASM was defined as “*failure of adequate trials of 2 tolerated, appropriately chosen and used antiepileptic drug schedules (whether as monotherapies or in combination) to achieve sustained seizure freedom*,”^21^, ^22^ whereas dogs with IE that were ASM‐responsive were characterized as “*patients with complete freedom of seizures or extension of the inter‐seizure interval to 3 times the longest pretreatment inter‐seizure interval and at least 3 months*.”^22^

### 2.2 CSF and serum

Paired CSF and serum samples were collected from all dogs during diagnostic evaluation with consent of their owners. Cerebrospinal fluid was obtained by atlanto‐occipital puncture with the dog in lateral recumbency during general anesthesia and examined within 30 minutes for total protein concentration (TP), total nucleated cell count (TNCC), and cytology. Normal CSF was defined as having a TP < 0.33 g/L, TNCC < 5/μL and unremarkable cytology findings. Blood samples were taken at the same time by peripheral venipuncture and centrifuged. Both CSF and serum samples were frozen within 1 hour after sampling and stored in plastic tubes at −80°C. Immunoglobulin G concentrations in both CSF and serum samples were measured using a IgG ELISA kit designed for dogs (Abcam, Cambridge, United Kingdom). Samples then were aliquoted and stored at −80°C until analysis of OCBs.

### 2.3 Detection of OCBs

**FIGURE 1:**
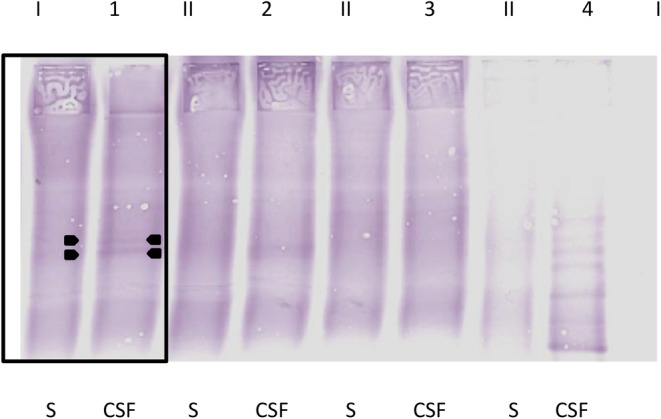
Immunoblot of 3 serum and CSF samples (nos. 1‐3) of dogs with idiopathic epilepsy (IE) and a human MS patient (no. 4) after isoelectric focusing. Dog no. 1 shows clear CSF‐specific OCBs. CSF, cerebrospinal fluid; S, serum. The samples of the human MS patient served as positive controls.

Detection of OCBs was performed in paired serum and CSF samples by isoelectric focusing (IEF) and immunoblotting (SEBIA Swiss GmBH, Wollerau, Switzerland) at the laboratory of the Department of Neurology, University Hospital Zurich as previously described.^23^ The gel containing fractionated proteins was transferred to a membrane and a canine goat‐anti‐IgG antibody (Bio‐Rad, Cressier, Switzerland; H + L, purified, polyclonal, Alkaline Phosphatase conjugated, product code AAI50A) was applied for the detection of OCBs. The immunoblots were independently evaluated by 2 experienced OCB raters. The criteria used in human medicine for description of the different OCB patterns was applied to characterize OCBs in the immunoblots of dogs.^24^ Cerebrospinal fluid‐specific OCBs were defined as ≥2 OCBs detected in CSF (Figure 1) but absent in the corresponding serum, as counterparts to the described type 2 and type 3 OCB patterns in human medicine.^24^

### 2.4 Statistics

Information on the dogs' signalment, sex, weight, age at first seizure, seizure type (focal, generalized, focal with secondary generalization), seizure severity (isolated, cluster or status epilepticus), medications received, age at CSF collection, epilepsy duration at time of CSF collection, time from last seizure to blood and CSF collection, number of seizures 3 months before blood and CSF collection and number of seizures 3 months after blood and CSF collection was gathered. Statistical analysis was carried out using NCSS (NCSS 12 Statistical Software: LLC, Kaysville, Utah, United States). Frequency of CSF‐specific OCBs was quantified as proportions in patients with and without CSF‐specific OCBs. The association between the presence of OCBs and other clinical variables including age at first seizure, seizure type, seizure severity, number of ASM and ASM response, time between last seizure day and CSF collection, age at CSF collection, and epilepsy duration at CSF collection was investigated using odds ratios and logistic regression models.

## 3 RESULTS

### 3.1 Study population

Eighty‐four dogs with IE were recruited from the Division of Clinical Neurology, Small Animal Clinic, Vetsuisse Faculty, University of Bern, Switzerland (n = 43), the Veterinary Practice Dr. A. Bathen‐Nöthen, Cologne, Germany (n = 27), and the Department of Small Animal Medicine and Surgery, University of Veterinary Medicine Hannover, Germany (n = 14). Twenty‐eight (33.3%) dogs with IE were categorized as ASM‐resistant and 56 (66.7%) dogs with IE as ASM‐responsive.

The study population consisted of 13/84 intact female, 18/84 spayed female, 31/84 intact male, and 22/84 neutered male dogs. Mean body weight was 23.7 kg (range, 2.1‐58 kg). Mean age at first seizure was 2.9 years (range, 0.5‐6 years), and mean age at CSF collection was 3.8 years (range, 1‐9 years). Mean epilepsy duration at CSF collection was 12 months (range, 0‐60 months), mean time from last seizure to blood and CSF collection was 43 days (range, 0‐1080 days).

The frequency of CSF‐specific OCBs among all dogs with IE was 15.5% (95% confidence interval [CI], 8.5%‐25%; 13/84 dogs).

### 3.2 Comparison of clinical features in IE dogs with and without CSF‐specific OCBs

Dogs with IE and detection of CSF‐specific OCBs seemed to show a breed predisposition when compared with the group of dogs with no CSF‐specific OCBs, as evidence for an association between the Australian Shepherd breed and presence of OCBs was detected (odds ratio, 4.82; 95% CI, 1.14‐20.41; *P* = .03; Table 1). No evidence of an association between the presence of CSF‐specific OCBs and ASM response, seizure type, seizure severity or number of ASMs was found. Regarding seizure severity, dogs with IE and CSF‐specific OCBs also had cluster seizures (84.6% vs 64.8%; Table 1), but with lack of evidence (odds ratio, 1.91; 95% CI, 0.38‐9.57; *P* = .43). Moreover, no evidence was obtained for CSF‐specific OCBs to be associated with age at first seizure, seizure type, seizure severity, number of ASMs, time between the last seizure day and CSF collection, age at CSF collection, and epilepsy duration at CSF collection (Tables 2 and 3).

**TABLE 1:** Number of dogs with IE in relation to presence or absence of CSF‐specific OCBs.

		Dogs with CSF‐specific OCBs (N = 13)	Dogs with no CSF‐specific OCBs (N = 71)
		N = (%)	N = (%)
Breed		4 (30.8%) Australian Shepherds 3 (23.0%) Siberian Huskies 1 (7.7%) Mixed breed 1 (7.7%) Alaskan Malamute, French Bulldog, Golden Retriever, Biewer Terrier, Miniature Poodle	12 (16.9%) Mixed breeds 6 (8.5%) Australian Shepherds 4 (5.6%) French Bulldogs 4 (5.6%) Labrador Retrievers 3 (4.2%) Golden Retrievers 2 (2.8%) American Staffordshire Terriers 2 (2.8%) English Springer Spaniels 2 (2.8%) Great Danes 2 (2.8%) Miniature Pinchers 2 (2.8%) Pugs 2 (2.8%) Boxers 30 (42.4%) Others^a^
Gender	Male intact	5 (38.4%)	26 (36.6%)
	Male neutered	3 (23.1%)	19 (26.8%)
	Female intact	4 (30.8%)	9 (12.7%)
	Female spayed	1 (7.7%)	17 (23.9%)
Treatment response to ASM	ASM‐responsive	7 (53.9%)	49 (69.0%)
	ASM‐resistant	6 (46.1%)	22 (31.0%)
Seizure type	Generalized seizures only	8 (61.5%)	49 (69.0%)
	Focal and/or generalized seizures	5 (38.5%)	22 (31.0%)
Seizure severity	Only isolated seizures	2 (15.4%)	16 (22.5%)
	Cluster seizures	11 (84.6%)	46 (64.8%)
	Status epilepticus and cluster seizures	0 (0%)	9 (12.7%)
Number of ASMs	0 or 1 ASM	8 (61.6%)	42 (59.2%)
	2 or 3 ASMs	5 (38.4%)	29 (40.8%)

Abbreviation: ASM, anti‐seizure medication.

^a^ Others include Border Collie, Westphalian Dachsbracke, Irish Setter, Golden Setter, Perro sin pelo del Perú, American Bulldog, Curly‐coated Retriever, Dobermann, Pekingese, Beagle, Miniature Schnauzer, Miniature Australian Shepherd, Dutch Shepherd, Belgian Shepherd, Shih Tzu, Giant Schnauzer, Irish Water Spaniel, Yorkshire Terrier, Boston Terrier, Chihuahua, Poodle, Elo, Cane Corso Italiano, Briard, Pomeranian, Rhodesian Ridgeback, Bullmastiff, Eurasian, Australian Labradoodle, Float‐coated Retriever.

**TABLE 2:** Mean ± SD, median and IQR range in dogs with IE with CSF‐specific OCBs and dogs with no CSF‐specific OCBs.

Mean ± SD and range	Dogs with CSF‐specific OCBs	Dogs no CSF‐specific OCBs
	N = 13	N = 71
Body weight (in kg)	22.6 ± 12.0 (6.0‐50.2)	23.9 ± 12.8 (2.1‐58)
Age at first seizure (in years)	2.5 ± 1.6 (0.6‐6.0)	3.0 ± 1.5 (0.5‐6.0)
Age at CSF tap (in years)	3.6 ± 1.5 (1.0‐6.0)	3.8 ± 2.0 (1.0‐9.0)
Epilepsy duration at CSF tap (in months)	Median 2 (95% CL 1‐24)	Median 9 (95% CL 4‐12)
Time span from last seizure to CSF tap (in days)	Median 10.5 (95% CL 2‐30)	Median 14 (95% CL 10‐25)
Seizure frequency before treatment/3M	Median 6 (95% CL 3‐8)	Median 5 (95% CL 4‐6)
Seizure frequency after initiation of treatment/3M	Median 1 (95% CL 0‐8)	Median 1 (95% CL 0‐3)

Abbreviation: CL, confidence level.

**TABLE 3:** Characterization of dogs with IE and CSF‐specific OCBs.

	Breed	Gender	Body weight (in kg)	Age at first seizure (in years)	Age at CSF tap (in years)	Epilepsy duration at CSF tap (in months)	Time from last seizure to CSF tap (in days)	Total protein in CSF (g/L)
1	Siberian Husky	Male	38.2	0.6	3	24	2	0.3
2	Mixed breed	Female spayed	15.9	1	1	0.1	3	0.13
3	Australian Shepherd	Male	17.6	1	4	24	1	0.1
4	Biewer Terrier	Male neutered	6.5	4	6	48	5	0.25
5	Australian Shepherd	Female	21.4	1	2	12	30	0.24
6	French Bulldog	Female	18.2	1	3	24	n.k.	0.09
7	Siberian Husky	Male	19.7	2	2	1	16	0.16
8	Alaskan Malamute	Male	50.2	4	4	1	20	0.3
9	Australian Shepherd	Male neutered	22.7	3	5	2	40	0.3
10	Miniature Poodle	Male	6	6	6	1	20	0.1
11	Siberian Husky	Female	19.6	3	3	0.1	3	0.09
12	Australian Shepherd	Male neutered	28.4	3	4	1	1	0.14
13	Golden Retriever	Female	29.5	3	4	12	30	0.19

Abbreviations: CSF, cerebrospinal fluid; n.k., not known.

### 3.3 Dogs with IE and CSF‐specific OCBs

Among dogs with CSF‐specific OCBs, those with IE that were resistant to ASM had a longer epilepsy duration at the time of CSF collection (median, 24 months; range, 0.1‐48 months; Table 4) compared with ASM‐responsive dogs (median, 1 month; range, 0.1‐12 months), except 1 dog with an acute onset of cluster seizures that was euthanized 4 months later because of uncontrolled seizures. All dogs with ASM‐resistant IE were presented with cluster seizures. In addition to recurrent seizures, 2 dogs with IE and CSF‐specific OCBs manifested movement disorders with limb or truncal dyskinesia, and 1 dog had interictal aggression.

**TABLE 4:** Characterization of seizure characteristics and treatment response in dogs with IE and CSF‐specific OCBs.

	Seizure type	Seizure severity	Seizure frequency before ASM/3M	Seizure frequency after initiation of ASM/3M	Treatment response	Number of ASMs	Remarks
1	Generalized	Cluster	6	4	ASM‐resistant	3	
2	Generalized	Cluster	7	6	ASM‐resistant	1	
3	Generalized	Cluster	6	8	ASM‐resistant	2	
4	Focal & generalized	Cluster	6	9	ASM‐resistant	2	
5	Focal & generalized	Cluster	11	8	ASM‐resistant	3	
6	Focal & generalized	Cluster	9	12	ASM‐resistant	3	
7	Generalized	Cluster	5	1	ASM‐responsive	1	
8	Generalized	Isolated	2	0	ASM‐responsive	1	
9	Generalized	Cluster	6	0	ASM‐responsive	1	
10	Focal & generalized	Cluster	3	0	ASM‐responsive	0	Starts with stumbling and falling to the side
11	Generalized	Cluster	8	0	ASM‐responsive	1	Interictal aggression
12	Generalized	Cluster	3	0	ASM‐responsive	1	
13	Focal	Isolated	1	0	ASM‐responsive	1	Episodes of inability to move at night, with urination and defecation

Abbreviation: ASM, anti‐seizure medication.

## 4 DISCUSSION

Idiopathic epilepsy is the most common diagnosis in dogs presented with recurrent epileptic seizures, especially when between 6 months and 6 years of age.^25^ Seizure control can be challenging with approximately 33% of the dogs not responding adequately to ASM, where there is <50% decrease in seizures.^3^ Several factors are assumed to play a role in resistance to ASM, such as seizure frequency and duration, genetic background and alterations in epigenetic mechanisms, and neuroinflammation.^26^, ^27^ Furthermore, a subgroup of these dogs may experience pseudoresistance because of misdiagnosis, therapeutic errors or poor owner compliance.^28^ The underlying mechanisms, however, have not been fully elucidated. In human medicine, it is estimated that autoimmune epilepsy accounts for 6%‐37% of epilepsy of unknown etiology^29^ and 1% of epilepsy in total.^30^ Different neural antibodies have been described in human patients with epilepsy, and those with autoimmune encephalitis are more likely to go into remission with early implementation of additional immunomodulatory treatment.^31^, ^32^ In our study, the intrathecal immune reaction in dogs diagnosed with IE was assessed to investigate a potential underlying role of neuroinflammation. We found that 15.5% of dogs with IE had CSF‐specific OCBs. This frequency is comparable to the reported numbers of CSF‐specific OCBs in human epileptic patients (8.7%‐14%).^18^, ^33^ In contrast, CSF‐specific OCBs were mostly detected in dogs with meningoencephalitis of unknown origin (57%), followed by intracranial neoplasia (22%), steroid‐responsive meningitis‐arteritis (15%), intervertebral disc disease (13%), and IE (6%) in a previous study.^23^

Oligoclonal bands are strong, but non‐specific markers of chronic CNS inflammation.^34^, ^35^ Recent studies in humans suggest that 5%‐8% of cases with epilepsy of unknown etiology show intrathecal immunoglobulin synthesis.^36^, ^37^, ^38^ However, it is unclear whether increased intrathecal immunoglobulin synthesis implies the presence of autoimmune encephalitis, generalized immunoreaction, or seizure‐induced CSF changes.^36^, ^37^ Therefore, in human medicine additional variables are considered, such as presence of neural autoantibodies in the CSF or serum, to further corroborate an underlying immune‐mediated mechanism. In recent studies on human patients with ASM‐resistant epilepsy of unknown etiology, neural antibodies had a global pooled prevalence of 7.6% (95% CI, 4.6‐11.2) in serum or CSF or both.^7^ Seizures of autoimmune etiology usually are characterized by acute onset, high frequency, possible behavioral changes, and ASM resistance.^39^ In our study, the majority of dogs with CSF‐specific OCBs had cluster seizures. However, immunosuppressive treatment is not required for satisfactory seizure control in all seizure patients presenting with autoantibodies.^40^, ^41^, ^42^ Clinical features rather than presence of neural antibodies should guide implementation of immunotherapy.^40^ Characteristic neuropsychological symptoms of mesial temporal lobe deficits in humans such as memory loss or executive function impairment unfortunately are difficult to evaluate in dogs.^43^ In our study, 1 dog with CSF‐specific OCBs showed interictal aggression in addition to seizures. The human antibody prevalence in epilepsy (APE) score^5^ may be adopted as a recruitment criterion for dogs with epilepsy suspected to be autoimmune origin for future studies. Furthermore, a distinction has been made between acute symptomatic seizures secondary to autoimmune encephalitis or autoimmune‐associated epilepsy in human medicine. The former etiology of seizures is related to encephalitis caused by antibodies against extracellular neural synaptic and cell membrane antigens such as NMDAR,^11^ LGI1,^14^ GABA_B_R,^12^ and GABA_A_R^13^ or contactin‐associated protein‐like receptor 2 (CASPR2).^44^ When concomitant neoplasia has been excluded, patients with seizures secondary to this category of autoimmune encephalitis can achieve seizure freedom after adequate treatment.^39^ In contrast, patients with autoimmune‐associated epilepsy produce antibodies against intracellular antigens (such as glutamic acid decarboxylase [GAD]). These antibodies are a by‐product of the cytotoxic T‐cell immune reaction, and immunotherapy usually proves to be ineffective. Studies on the role of neuroinflammation and occurrence of neural antibodies in dogs are warranted to differentiate types of autoimmune‐related seizures and provide more guidance regarding treatment and prognosis.

Australian Shepherds and Siberian Huskies are known to be among the dog breeds more prone to ASM‐resistant seizures, and a genetic background is suspected.^2^, ^45^ In our study population, there were insufficient numbers of Siberian Huskies to test for a breed association. We also found evidence of association of the presence of CSF‐specific OCBs in Australian Shepherds. In humans, it is known that there is a known genetic predisposition for neural antibody production.^46^, ^47^, ^48^ However, detection of neural antibodies in dogs with epilepsy has not been successful thus so far,^49^ and needs to be investigated in dogs in general and especially in certain dog breeds.

We showed that CSF‐specific OCBs are present in a subgroup of dogs with IE. These findings suggest that intrathecal IgG synthesis as a sign of neuroinflammation may play a role in disease pathogenesis. Additional studies are warranted to investigate the role of intrathecal IgG synthesis in ASM resistance in dogs with IE. The potential role of neuroinflammation in IE indicates that research into personalized medicine approaches in dogs with epilepsy, with a specific focus on neuroinflammation, could offer opportunities for the development of innovative treatment modalities and refinement of existing protocols.

## CONFLICT OF INTEREST DECLARATION

Authors declare no conflict of interest.

## OFF‐LABEL ANTIMICROBIAL DECLARATION

Authors declare no off‐label use of antimicrobials.

## INSTITUTIONAL ANIMAL CARE AND USE COMMITTEE (IACUC) OR OTHER APPROVAL DECLARATION

Authors declare no IACUC or other approval was needed.

## HUMAN ETHICS APPROVAL DECLARATION

Authors declare human ethics approval was not needed for this study.
